# Limiting spread of VIM-positive *Pseudomonas aeruginosa* from colonized sink drains in a tertiary care hospital: A before-and-after study

**DOI:** 10.1371/journal.pone.0282090

**Published:** 2023-03-24

**Authors:** Jannette Pirzadian, Anne F. Voor in ‘t holt, Mehjabeen Hossain, Corné H. W. Klaassen, Inge de Goeij, Heidy H. H. T. Koene, Lonneke G. M. Bode, Margreet C. Vos, Juliëtte A. Severin

**Affiliations:** Department of Medical Microbiology and Infectious Diseases, Erasmus MC University Medical Center Rotterdam, Rotterdam, The Netherlands; Amphia Ziekenhuis, NETHERLANDS

## Abstract

**Background:**

In healthcare environments, sinks are being increasingly recognized as reservoirs for multidrug-resistant Gram-negative bacteria. In our hospital, carbapenemase-producing, Verona Integron-encoded Metallo-beta-lactamase (VIM)-positive *Pseudomonas aeruginosa* (VIM-PA) was detected at low endemicity in patients, and environmental culturing revealed that sink drains were primary reservoirs. Therefore, an intervention was initiated in several wards to install sink drain plugs as physical barriers against splashing to prevent transmission of VIM-PA from drain reservoirs to the surrounding sink environment.

**Aim:**

To assess the efficacy of the intervention on limiting spread of VIM-PA.

**Methods:**

Swabs were taken from inner sink environments (*i*.*e*. drains), and outer sink environments (*i*.*e*. wash basins, faucet aerators, and countertops) twice before and three times after the intervention. Siphon water and drain wells were also sampled before and at the moment of the intervention, respectively. All samples were screened for VIM-PA, and isolates were typed with multiple-locus variable-number tandem repeat analysis (MLVA).

**Results:**

There was a significant reduction in VIM-PA positivity in both inner (*P*-value <0.001) and outer (*P*-value 0.001) sink environments after the intervention. However, VIM-PA recolonization was observed in the inner sink environments of patient rooms, and also in rooms exclusive to healthcare personnel, over time. Surfaces in the outer sink environment were rarely positive for VIM-PA after the intervention. MLVA revealed three genetic clusters, with one found in all wards and room types during the study period.

**Conclusions:**

Drain plugs are a simple and effective infection prevention and control measure to contain spread of VIM-PA from drain reservoirs.

## Introduction

In healthcare environments, sinks and wastewater pipelines are being increasingly recognized as reservoirs for clinically-relevant and multidrug-resistant Gram-negative bacteria, such as *Pseudomonas aeruginosa*, *Klebsiella pneumoniae*, and *Acinetobacter baumannii* [1–3]. These reservoirs may contribute to nosocomial infections when contaminated droplets or aerosols disperse from sink drains onto surrounding surfaces in the innate patient environment [4–6]. Furthermore, these reservoirs are difficult to eradicate due to biofilm formation within the lumen of pipelines [4,7,8]. Chemical interventions are typically ineffective at removing biofilms in the long-term, since biofilm structures show reduced permeability to biocides, permitting the regrowth of surviving cells in as little as one week after disinfection [5,7–9]. A variety of physical interventions have been employed to end outbreaks by biofilm-associated pathogens, such as the replacement of contaminated sinks/plumbing and the use of heating and vibrational devices to disinfect wastewater in the siphon base; however, drains are often recolonized as sinks are exposed to contaminated patient materials or from retrograde growth from p-traps [10–16].

At the Erasmus MC University Medical Center (Erasmus MC), environmental sampling for carbapenemase-producing, Verona Integron-encoded Metallo-beta-lactamase (VIM)-positive *P*. *aeruginosa* (VIM-PA) was initiated in 2011 in response to suspected nosocomial transmission. It was revealed that in certain wards, sink drains were persistently colonized by VIM-PA, and the most prevalent environmental clones belonged to high-risk sequence type (ST) 111 [17]. ST111 clones have contributed to the successful spread of antibiotic-resistant lineages worldwide [18–20]. In our setting, multiple interventional strategies have been performed to reduce the environmental burden, such as complete sink/drain replacement and disinfection using chlorine and steam, but these were all unsuccessful at preventing recolonization. Therefore, in 2013, we initiated an intervention in several wards in which sink drain plugs were installed to act as physical barriers against splashing. We hypothesized that limiting the dispersal of droplets carrying VIM-PA from the sink drain to the surrounding environment would reduce transmission from sinks to patients. In this study, the efficacy of this intervention on limiting spread to the surrounding sink environment was assessed using epidemiological and molecular approaches.

## Material and methods

### Setting

The Erasmus MC is a tertiary care hospital in Rotterdam, the Netherlands. At the time of the study, the hospital contained 1,200 beds, and was organized into 48 wards covering all medical specialties. Adult intensive care units (ICUs) comprised only single-occupancy patient rooms and patient isolation rooms, while the other wards comprised single- to multiple-occupancy (*i*.*e*. 2-, 3- or 4-bed) patient rooms. Six wards most affected by VIM-PA sink colonization were subjected to the intervention: two adult ICU wards (designated ICU-1 and ICU-2), two surgical wards (designated Surgery-1 and Surgery-2), Gastroenterology and Hepatology, and Pulmonology. Surgery-1 mainly admitted patients undergoing digestive tract surgery, and Surgery-2 mainly admitted patients undergoing vascular surgery or renal transplantation. The following room types were included in one or more of the wards: single-occupancy patient rooms; single-occupancy patient isolation rooms; anterooms of single-occupancy patient isolation rooms; laboratories; dirty utility rooms; patient bathrooms containing a bathtub, shower, and/or toilet; anterooms of bathrooms containing two toilets and two showers; medication rooms; staff kitchens; storage rooms; 4-bed patient rooms; 2-bed patient rooms; nursing posts; and meeting rooms. All isolation rooms contained an anteroom with a sink.

### Study design

**Fig 1:**
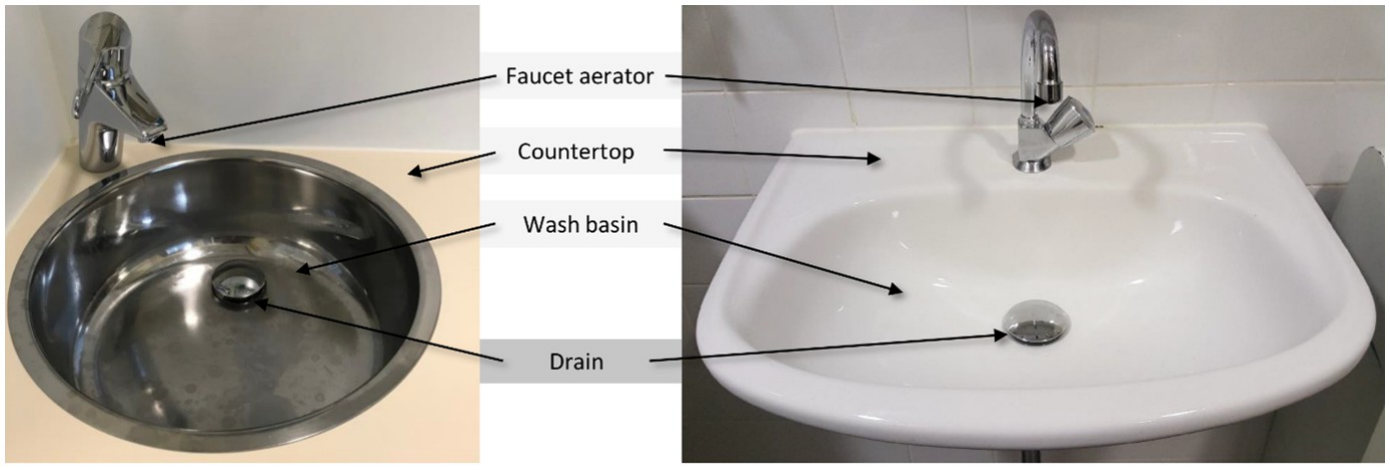
Sink surfaces cultured during every sampling moment. Shown are the sites that were swabbed during every sampling moment. The drain plugs that were installed are shown covering the drains of two different sinks included in the intervention. The inner sink environment comprised the drain (dark grey), and the outer sink environment comprised the faucet aerator, countertop, and wash basin (light grey). Drain wells and p-traps/siphon water were also part of the inner sink environment, but these are not shown in the figure.

This was a prospective before-and-after study conducted between January 2013 and June 2014. In this study, environmental sampling was performed before, after, and at the moment of the intervention. The intervention entailed removing the existing sink strainer and drain well from all sinks in the ward, and replacing them with a new sink strainer that included a chrome plated brass and stainless steel drain plug without holes (article number UD.526.51, Raminex, Utrecht, the Netherlands) (Fig 1). The drain plug cost, without taxes, around 50 euros each. After this, the countertop and surrounding walls, wash basin, and new drain plug were cleaned (in this order) using a microfiber cloth, after which 6 L 250 ppm chlorine were carefully poured into the drain to disinfect the pipelines. Plumbing and cleaning personnel used gowns and gloves during these activities, and all utensils were cleaned and disinfected between sinks. The intervention was executed between July and September 2013, and only performed in rooms when no patients were present.

The pre-intervention phase began in January 2013, and was completed in September 2013, or until sink drain plugs were installed in all sinks of the ward. During the pre-intervention phase, three rounds of environmental sampling were performed: one round between eight and nine months before the intervention, one round between two weeks and three months before the intervention, and one round at the moment of the intervention.

The post-intervention phase included three rounds of environmental sampling: one round a month after the intervention, one round two months after the intervention, and one round six months after the intervention.

### Sink cultures

The following sink surfaces were tested during each sampling moment: the countertop, wash basin, faucet aerator, and drain (Fig 1). In the pre-intervention phase, no drain plugs existed, so swabs were directly inserted into drains or between the holes of sink strainers. In the post-intervention phase, swabs were inserted underneath drain plugs and into the drains. Samples were taken with sterile cotton-tipped swabs, transported in Amies medium (BBL CultureSwab Plus, Copan Italia, Brescia, Italy), and incubated overnight at 35°C in 5 mL tryptic soy broth with 2 mg/L ceftazidime and 50 mg/L vancomycin. DNA was extracted from this broth using the MagNa Pure 96 DNA and Viral Nucleic Acid Small Volume Kit (Roche Diagnostics, Almere, the Netherlands), and then screened for the *bla*_VIM_ gene using a previously-described PCR protocol [21]. Broths with a positive PCR result were subcultured onto Tryptic Soy Agar II with 5% Sheep Blood (TSA) (BD Diagnostics, Breda, the Netherlands). Bacteria were identified using MALDI-TOF MS (Bruker Daltonik, Bremen, Germany), and *P*. *aeruginosa* were subjected to *bla*_VIM_ PCR. All confirmed VIM-PA isolates were stored at -80°C.

In the wards ICU-1 and Gastroenterology and Hepatology, sinks in three rooms each (six rooms total) were also screened before the intervention at different time points for a time point analysis to study if cleaning habits influenced culture results. In ICU-1, these rooms consisted of two single-occupancy patient rooms and one laboratory, and in Gastroenterology and Hepatology, these consisted of one medication room, one 4-bed patient room, and one 2-bed patient room. The countertop, washbasin, faucet aerator, drain, and siphon water were screened for *bla*_VIM_ for five consecutive days at 9:00, 12:30, and 16:00. Cleaning staff was asked to register the time when the sink was cleaned.

Only in the pre-intervention phase, siphon water samples were also taken from p-traps of all sinks using a sterile female urinary catheter and a 50 mL syringe, and collected in a sterile tube. In the laboratory, a sterile cotton-tipped swab was soaked in the water sample, and incubated in broth as described above. Only at the moment of the intervention, the drain wells of all sinks were also sterilely transported to the laboratory, where they were sampled using a sterile cotton-tipped swab, and incubated in broth as described above.

### Patient cultures

Patients with a positive culture for VIM-PA between February 2013 and February 2014, and who were admitted to one or more of the participating wards, were also included to identify possible transmission events. Patient cultures were obtained and characterized as previously described [22]. Approval to conduct the study was received from the medical ethical research committee of the Erasmus MC (MEC-2015-306). Participation of patients is authorized through passive informed consent via electronic patient charts. Eligible patients are cross-checked with the opt-out list. Patients who did not allow that their data were to be used for research are not included in the study population.

### Clonal relatedness

All stored VIM-PA isolates were cultured on TSA. DNA was extracted from pure cultures using InstaGene Matrix (Bio-Rad Laboratories, Veenendaal, the Netherlands) according to the manufacturer’s instructions. Multiple-locus variable-number tandem repeat (VNTR) analysis (MLVA) was performed by amplifying eight loci, followed by capillary electrophoresis and analysis as previously described [23].

### Other infection prevention and control measures

Several other infection prevention and control (IPC) measures were implemented both before and during this study to halt environmental transmission of VIM-PA from contaminated sinks to patients on the ICU [22,24]. Firstly, in October 2010, all siphons and drains in ICU-1 patient rooms were replaced. Secondly, from February 2011 and onwards, it was emphasized to ICU healthcare personnel (HCP) that it was very important not to put clean materials in the wash basin and its surroundings. Finally, from December 2011 and onwards, usage of tap water was discontinued, and only bottled water was permitted for use.

### Definitions and statistical analysis

The inner sink environment was defined as siphon water, drain wells, and the drain. The outer sink environment was defined as the countertop, the wash basin, and the faucet aerator. The inner/outer sink environment (Fig 1) was considered positive if at least one of their surfaces tested positive for VIM-PA. For the time point analysis, data from each room and ward were first analyzed separately to study differences in, for example, cleaning habits. Then, data were combined to recognize trends. Since siphon water and drain wells were only sampled in the pre-intervention phase, data were analyzed both including and excluding siphon water; drain wells were only analyzed separately and not included in the before-and-after analysis. For both, data are designated in this study as occurring “before the intervention.” Sinks in one bathroom with a bath tub, 17 showers, three showers with toilets, six anterooms containing two toilets and two showers, and 11 toilets were grouped under the heading ‘Bathroom’. Two laboratories, one meeting room, one nursing post, two staff kitchens, and three storage rooms were grouped under the heading ‘HCP spaces’. Sinks in dirty utility rooms were assessed separately and not included in the before-and-after analysis, as these spaces were frequently exposed to contaminated patient materials and were expected to have higher chances of being positive for VIM-PA.

Data were presented as percentages, medians, or means. Differences before/after the intervention and between groups were identified using the Chi-square statistic in SPSS v25 (IBM Corp., Armonk, New York, USA). *P*-values <0.05 were considered statistically significant.

## Results

### VIM-PA in the sink environment before and after the intervention

In total, 150 sinks in 143 rooms were included in the before-and-after analysis. Due to missing data in the room types Bathroom and HCP spaces, for the overall analysis, 144 sinks were used to analyze both the inner and outer sink environments. In all room types, the outer sink environment was less frequently positive for VIM-PA compared to the inner sink environment both before and after the intervention (Table 1). Overall, there was a significant reduction in VIM-PA positivity in both the inner and outer sink environments after sink drain plugs were installed. In 12 sinks, only the siphon water was positive for VIM-PA before the intervention. When excluding siphon water from the inner sink environment, there was still a significant reduction in VIM-PA positivity (34.7% vs. 22.9%, P<0.001) in the inner sink environment after the intervention. Additionally, positivity went down in each room type after the intervention, except in anterooms of ICU single-occupancy patient isolation rooms (outer sink environment), though this effect was non-significant.

**Table 1:** Before-and-after analysis of VIM-PA cultured in the inner and outer sink environments. Percentages of VIM-PA-positive samples are shown per room type.

Room type	No. of sinks in rooms	Inner sink environment (%)		*P*-value	Outer sink environment (%)		*P*-value
		Before	After		Before	After	
Single-occupancy patient room	2	1 (50)	1 (50)	na	0 (0)	0 (0)	na
Single-occupancy patient isolation room	4^3^	2 (50)	1 (25)	na	1 (25)	0 (0)	na
Anteroom of single-occupancy patient isolation room	4^3^	0 (0)	0 (0)	na	0 (0)	0 (0)	na
2-bed patient room	35^4^	7 (20)	6 (17.1)	0.370	2 (5.7)	1 (2.9)	na
4-bed patient room^1^	12^5^	3 (25)	2 (16.7)	na	0 (0)	0 (0)	na
ICU single-occupancy patient room	26^6^	24 (92.3)	11 (42.3)	0.819	4 (15.4)	3 (11.5)	na
ICU single-occupancy patient isolation room	8^7^	8 (100)	6 (75)	na	3 (37.5)	3 (37.5)	na
Anteroom of ICU single-occupancy patient isolation room	8^8^	3 (37.5)	1 (12.5)	na	0 (0)	1 (12.5)	na
Bathroom^2^	38^9^	6 (16.7)	0 (0)	na	0 (0)	0 (0)	na
HCP spaces	9^10^	3 (37.5)	2 (33.3)	na	2 (25)	0 (0)	na
Medication room	4^11^	4 (100)	3 (75)	na	3 (75)	1 (25)	na
**Total**	**144** ^12^	**61 (42.4)**	**33 (22.9)**	**<0.001**	**15 (10.4)**	**9 (6.2)**	**0.001**

Abbreviations: ICU, intensive care unit; No., number; HCP, healthcare personnel; na, not applicable.

^1^Two rooms that had two sinks were included for a total of 12 sinks in 10 rooms.

^2^Five rooms that had two sinks were included for a total of 38 sinks in 33 rooms.

^3-11^For the number of rooms/time points where sampling was not performed, see S1 Text in S1 File.

^12^Sinks in six rooms were not sampled in either the inner or outer sink environment, and were excluded from the analysis.

### Increasing VIM-PA positivity after the intervention

One, two, and six months after the intervention, the sink environment was cultured. In the inner sink environment, VIM-PA positivity increased over time in 4-bed patient rooms, ICU single-occupancy patient rooms, HCP spaces, and medication rooms (Table 2). In one 4-bed patient room containing two sinks, one of the sinks became positive in month 2, and both sinks were positive in month 6. For ICU single-occupancy patient rooms, three sinks in three different rooms were positive in month 2, and nine sinks in nine rooms (including one sink that was also positive in month 2) were positive in month 6. For medication rooms, one sink was positive in months 1, 2 and 6; one additional sink became positive in month 2 and stayed positive in month 6; and one additional sink became positive in month 6. In the outer sink environment, cultures were rarely positive, and positivity did not increase over time. All raw data showing culture positivity per sampling moment per room type is available in S2 Table.

**Table 2:** Heat map showing the percentages of VIM-PA-positive samples at one, two, and six months after the intervention per room type.

Room type	No. of sinks	% positive inner sink environments			% positive outer sink environments		
		1M	2M	6M	1M	2M	6M
Single-occupancy patient room	2	50	50	50	0	0	0
Single-occupancy patient isolation room	4	0	0	25	0	0	0
Anteroom of single-occupancy patient isolation room	4	0	0	0	0	0	0
2-bed patient room	35	9^4^	9^1^	9	3^1^, ^5^	0	0
**4-bed patient room**	12	0^1^	8	17	0^1^	0	0
**ICU single-occupancy patient room**	26	0^1^	12	35	0^1^	8	4
ICU single-occupancy patient isolation room	8	50	57^1^	25	25	14	0
Anteroom of ICU single-occupancy patient isolation room	8	0	0	13	0	0	13
Bathroom	38	0^3^	0^1^	0	0^4^	0^1^	0
**HCP spaces**	9	0^4^	17^3^	20^2^	0^2^	0^3^	0^2^
**Medication room**	4	33^1^	67^1^	75	0^1^	0^1^	25

Abbreviations: M, months; HCP, healthcare personnel; ICU, intensive care unit; No., number.

^1^One sample missing.

^2^Four samples missing.

^3^Three samples missing.

^4^Two samples missing.

^5^One room.

Comparing sink surfaces, countertops and faucet aerators were found to be the least often positive for VIM-PA overall, and had no positive cultures after the intervention (Table 3, S3 Fig in S1 File). Therefore, any positive cultures found in the outer sink environment after the intervention were from wash basins; however, percentages of VIM-PA in wash basins after the intervention were low, with no increase observed over time. In drains, there was an overall increasing percentage of positive cultures after the intervention.

**Table 3:** Percentages of VIM-PA-positive samples found at every sampling moment before and after the intervention per sink surface.

Sink environment	Sink surface	Time point (%)					
		8M–9M before (n = 150)	2 weeks—3M before (n = 150)	Moment of intervention (n = 152)	1M after (n = 150)	2M after (n = 150)	6M after (n = 150)
Inner	Siphon	27 (19.9)^1^	34 (24.1)^2^	na	na	na	na
Inner	Drain well	na	na	30 (21.7)^1^	na	na	na
Inner	Drain	27 (19.7)^3^	30 (21.1)^4^	6 (4.5)^5^	9 (6.5)^6^	15 (10.5)^7^	23 (15.8)^8^
Outer	Countertop	0 (0)^3^	1 (0.7)^4^	2 (1.5)^5^	0 (0)^6^	0 (0)^7^	0 (0)^8^
Outer	Wash basin	7 (5.1)^3^	11 (7.7)^4^	2 (1.5)^5^	3 (2.2)^9^	3 (2.1)^7^	3 (2.1)^8^
Outer	Faucet aerator	1 (0.7)^3^	1 (0.7)^4^	0 (0)^10^	0 (0)^9^	0 (0)^7^	0 (0)^8^

Abbreviations: M, months; na, not applicable.

^1^14 samples missing.

^2^Nine samples missing.

^3^13 samples missing.

^4^Eight samples missing.

^5^19 samples missing.

^6^12 samples missing.

^7^Seven samples missing.

^8^Four samples missing.

^9^11 samples missing.

^10^29 samples missing.

### Removed wells

Thirty out of 138 (21.7%) removed wells tested positive for VIM-PA (Table 3). Most positive wells belonged to ICU single-occupancy patient rooms (*i*.*e*. 17 out of 33 removed wells, 51.5%). At nursing posts, laboratories, staff kitchens, and storage rooms, none of the removed wells tested positive for VIM-PA.

### Dirty utility rooms

In a separate analysis, twelve sinks in eight dirty utility rooms were screened for VIM-PA. Though non-significant, the number of drains that tested positive for VIM-PA also decreased after the intervention (*P*-value 0.155) (S4 Table in S1 File). While countertops and faucet aerators never tested positive after the intervention, three wash basins tested positive after the intervention. At the moment of the intervention, four out of 12 removed wells (33.3%) from dirty utility rooms tested positive for VIM-PA.

### Time point analysis

In sinks from four rooms (both single-occupancy patient rooms, the 2-bed patient room, and the medication room), the inner sink environment was positive on at least one out of five days (S5 Table in S1 File). In the outer sink environment, wash basins were the most frequently positive surface in these rooms. Only in one out of 90 (1.1%) times the six included countertops were tested was a countertop positive. Faucet aerators only tested positive in one room on one day (day 4) at two different time points. In both cases, these positive cultures were from the medication room. Sinks from two rooms, the laboratory and the 4-bed patient room, were always negative for VIM-PA.

There was a slight increase in positive drains during the day. Most wash basins were positive at 12:30h, with numbers decreasing in the morning and in the afternoon. Sinks and countertops were always cleaned between the 9:00h and 12:30h time points (*i*.*e*. earliest reported time 10:25h, latest reported time 12:00h), which could be associated with the increased positivity of wash basins at 12:30h.

### Patient samples

During the study, ten patients screened positive for VIM-PA colonization in the participating wards: before the intervention, three patients in ICU-1, one in ICU-2, and two in Surgery-1 tested positive, and after the intervention, two patients in ICU-1 and two in ICU-2 tested positive. All ten patients had been admitted to different rooms, primarily ICU single-occupancy patient rooms.

### Clonal relatedness

**Fig 2:**
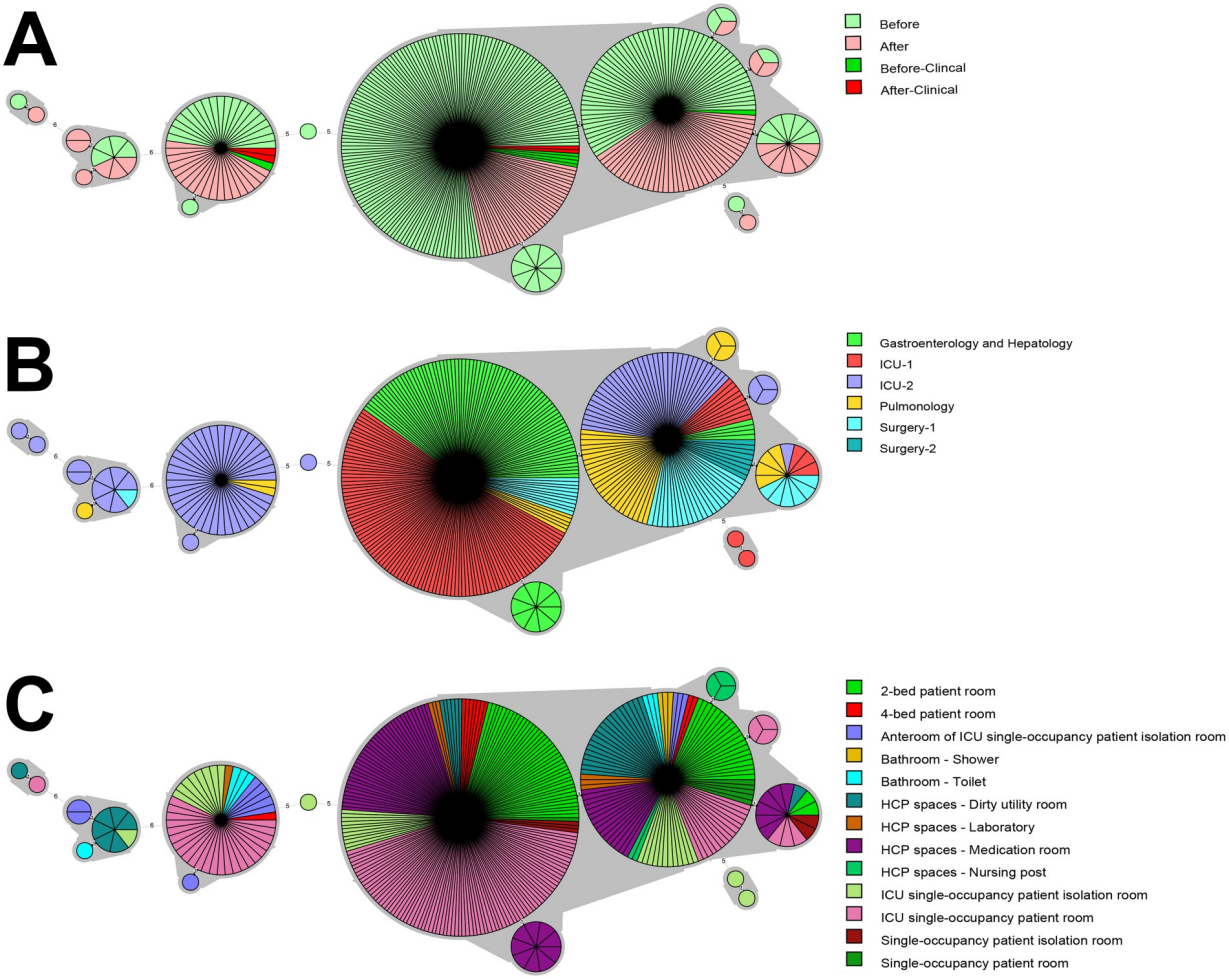
Minimum-spanning trees of VIM-PA isolates A) found before or after the intervention, and their B) ward of origin and C) room type, based on MLVA. Isolates found at the moment of the intervention are categorized as “before” isolates. Clinical isolates are indicated in panel A. Each circle represents a genotype, and depicts the number of isolates of that genotype. Numbers on connecting lines indicate the number of differing markers between genotypes. Gray shading indicates clusters of related genotypes differing in only a single MLVA marker.

All environmental and clinical VIM-PA isolates found both before and after the intervention were subjected to MLVA to determine clonal relatedness. Most isolates (327/385, 84.9%) contained high genotypic similarity and clustered together into what we have called Cluster 1 (Fig 2). Half of the isolates (194/385, 50%) contained an identical VNTR genotype, while several smaller clusters differed from this main cluster by only one MLVA marker. A smaller cluster of 42/385 (11%) isolates was designated Cluster 2. A third cluster, designated Cluster 3, contained 10/385 (3%) isolates.

All three genetic clusters were isolated from the environment both before and after the intervention (Fig 2A). Cluster 1 comprised environmental isolates from all participating wards (Fig 2B). Additionally, all clinical isolates found before or after the intervention belonged to Cluster 1 with the exception of three isolates (one found before, and two after); these isolates belonged to Cluster 2 (Fig 2A). Cluster 2 isolates dominated ICU-2, with the exception of two isolates from Pulmonology. Cluster 3 environmental isolates were found in ICU-2, Pulmonology, and Surgery-1; no clinical isolates belonged to Cluster 3. Concerning wards, environmental and clinical isolates from ICU-1 belonged exclusively to Cluster 1 with the exception of two incidental genotypes. Environmental isolates from Gastroenterology & Hepatology and Surgery-2 also only belonged to Cluster 1. Concerning room type, isolates from Cluster 1 were found in all room types (Fig 2C). Isolates from medication rooms and nursing posts belonged exclusively to Cluster 1. Though few isolates belonged to Cluster 3, this cluster mainly comprised isolates from dirty utility rooms.

## Discussion

### Summary of evidence

In this study, we observed that installing drain plugs in the sinks of six wards to act as physical barriers against splashing significantly limited spread of VIM-PA from the inner sink environment to the outer sink environment. VIM-PA positivity was significantly reduced in both the inner and outer sink environments after the intervention, though we observed increasing numbers of VIM-PA-positive sinks in the inner sink environment within six months. In the outer sink environment, surfaces rarely tested positive for VIM-PA after the intervention, with no increase observed at six months. Therefore, our findings reinforce previous reports that employing drain plugs/covers as splashing barriers is an effective and simple method for limiting dispersal of clinically-relevant pathogens from sink drains [25,26].

During drain plug installation, sink surfaces were cleaned and the inner sink environment was disinfected with chlorine, which could be one explanation for the reduction in VIM-PA seen one month after the intervention. However, the inner sink environment quickly became recolonized by VIM-PA within six months. VIM-PA positivity also increased in some room types over time, including in rooms restricted only to healthcare workers, such as medication rooms; since few VIM-PA-positive patients were identified during the study period that could have recolonized the sinks, this suggests that treatment with chlorine during the intervention was ineffective at eradicating existing VIM-PA reservoirs that were present in the pipelines. In previous studies, chlorine-containing solutions and other hospital-grade disinfectants were shown to be insufficient at eliminating VIM-PA colonization in sinks, but alternative disinfectants such as 24% acetic acid or probiotic cleansers could be more effective at eradicating *P*. *aeruginosa* and other pathogens in the long-term [21,27–29].

The outer sink environment was rarely positive for VIM-PA after the intervention. Only three positive cultures were identified from wash basins, and none from faucet aerators or countertops within the six-month follow-up. However, the time point analysis, which was performed before the intervention, revealed that wash basins were more often positive after cleaning. These results prompted an audit of the cleaning procedure and re-training of cleaning personnel in the ward Gastroenterology and Hepatology. While necessary for managing colonization, cleaning is considered a transmission risk when not performed correctly, as bacteria from the inner sink environment may be introduced to the outer sink environment. It is important to note that the presence of a drain plug in a sink provides another surface on which biofilm-forming microorganisms may grow; however, any growth on the top of the drain plug will be regularly wiped away when the wash basins are cleaned, so persistent growth will be limited to the underside of drain plugs. Therefore, since the inner sink environment (especially drains) will more often be a source of VIM-PA, limiting dispersal to the outer sink environment is an important consideration for IPC, and increasing amounts of VIM-PA in the inner sink environment continue posing a concern. Healthcare and cleaning personnel must be conscious of sink reservoirs, and focus on proper cleaning protocols. In other outbreak investigations, it was also proposed that reviewing cleaning protocols, educating staff on proper waste disposal, and instructing staff to report problems with sink drainage may all help avoid contamination of the outer sink environment [12,30]. Additionally, we previously observed that other factors, such as faucet position relative to the drain and dripping from the faucet, influenced the incidence and distance that dispersed droplets could be found from the drain; even in these cases, drain plugs significantly reduced dispersal to the outer sink environment [31].

A surprising observation was how many sinks in HCP spaces were colonized by VIM-PA. Unlike dirty utility rooms, medication rooms are expected to be clean working spaces. However, the disposal of medications, enteral feeding, and antibiotics down sink drains in these rooms may offer a source of enrichment to pathogenic bacteria. Conversely, it is expected that dirty utility rooms will harbor pathogenic bacteria, as HCP frequently handle contaminated patient materials in these spaces. Before the intervention, a high percentage of sinks in dirty utility rooms were found to be colonized by VIM-PA in the inner sink environment at our institution. After the intervention, VIM-PA positivity decreased in the inner sink environment, though not significantly. In the outer sink environment, VIM-PA was detected in three wash basins after the intervention, but a contaminating source from outside of the sink cannot be ruled out.

As an alternative interventional strategy, other studies have reported a significant reduction in patient colonization by Gram-negative bacilli after completely removing sinks from patient rooms and promoting “water-free” patient care [32,33]. However, sinks in HCP spaces have been found to have significantly high concentrations of *P*. *aeruginosa* in drains, siphons, and p-trap water [34]. Likewise, our study demonstrated that sinks in HCP-only spaces may be frequent sources of contamination, so removing sinks from patient rooms will have limited efficacy if contaminated sinks remain in other room types, permitting the transmission of pathogenic bacteria to patient spaces on the hands of HCP and other contaminated materials. Additionally, waterless patient care may not be feasible in all healthcare settings, since patients and healthcare personnel still require access to showers/sinks for personal hygiene and handwashing.

VIM-PA isolates obtained during the study period primarily belonged to three genetic clusters. From previous studies, it was known that the VNTR type to which Cluster 1 isolates belong is the most dominant genotype of environmental and clinical VIM-PA found in our hospital, and belongs to high-risk, epidemic lineage ST111, or serotype O12 [17]. This study reinforced that Cluster 1 isolates had likely spread throughout our hospital, since Cluster 1 isolates were recovered from sinks before/after the intervention and from all participating wards and room types, though transmission to patients was low. As ST111/O12 clones are associated with serious hospital-acquired infections, and are often multidrug resistant, it is of particular interest that these isolates are monitored and transmission is controlled [17,18].

### Limitations and strengths

This study had some limitations. Firstly, this was a single-center study. Secondly, patients in adult ICUs were screened twice weekly for VIM-PA, but patients in other wards were not screened regularly, so positive patients may have been missed. However, the frequency of patient sampling remained the same both before and after the intervention. Thirdly, the number of samples per sink that were taken at the moment of the intervention and after the intervention was low; additionally, sampling could not be performed in some rooms and/or during some sampling moments due to the presence of a sick patient, so there were missing samples. Fourthly, sink design differences were not investigated, so it is not possible to draw conclusions based on different sink materials, shapes, designs, or whether the surface smoothness of sinks affected the level of attrition. Finally, clonal relatedness of isolates was assessed using MLVA for eight repeat regions. Implementing a whole-genome sequencing approach could compare the three major clusters with higher resolution, and aid in outbreak investigations; this will be assessed in future studies. Nevertheless, the strengths of our study were that the intervention was implemented on hospital wards, a large number of patient rooms and HCP spaces could be included, and transmission to patients and the outer sink environment could be tracked. In contrast with other studies investigating the impact of sink drains/covers, our investigation included multiple wards (not only ICUs) and a six-month follow-up. In some room types, we observed increasing VIM-PA positivity in the inner sink environment within six months after the intervention, so in future studies, it would be interesting to continue tracking positivity for longer periods to observe if surfaces in the outer sink environment begin testing positively over time. Furthermore, at the start of the study, the VIM-PA outbreak had been halted and few patients had screened positive, so the impact of environmental reservoirs on transmission could be assessed during a low-endemic setting.

### Conclusions

Installing sink drain plugs was an effective measure at preventing transmission of VIM-PA from the inner to the outer sink environment. Though VIM-PA recolonization occurred in the inner sink environment in the six months following the intervention, no increase in VIM-PA positivity could be seen in the outer sink environment. Therefore, multidrug-resistant pathogens such as VIM-PA can be contained within inner sink environments, but transmission to the outer sink environment should be routinely monitored, as the effect of sink material and design is unknown.

## Supporting information

S1 Table All environmental culture data per sampling moment per room type. In the column Time Point, -9 to -8, sample was taken 8–9 months before the intervention; -3 to -0.5, sample was taken between 2 weeks and 3 months before the intervention; 0, sample was taken at the moment of the intervention; 1, sample was taken 1 month after the intervention; 2, sample was taken 2 months after the intervention; 6, sample was taken 6 months after the intervention. In the columns under Environmental Sampling Outcomes, 1, surface positive for VIM-PA; 0, surface negative for VIM-PA; 999, missing sample.
(XLSX)

S2 Table All environmental culture data per sampling moment per room type. In the column Time Point, -9 to -8, sample was taken 8–9 months before the intervention; -3 to -0.5, sample was taken between 2 weeks and 3 months before the intervention; 0, sample was taken at the moment of the intervention; 1, sample was taken 1 month after the intervention; 2, sample was taken 2 months after the intervention; 6, sample was taken 6 months after the intervention. In the columns under Environmental Sampling Outcomes, 1, surface positive for VIM-PA; 0, surface negative for VIM-PA; 999, missing sample.
(XLSX)

S1 File Contains all supporting figures/tables with accompanying captions. (DOCX)
