# Supplementary material for: Limiting spread of VIM-positive Pseudomonas aeruginosa from colonized sink drains in a tertiary care hospital: A before-and-after study
Source: PLoS One. 2023 Mar 24;18(3):e0282090. doi: 10.1371/journal.pone.0282090 (PMC10038242; doi:10.1371/journal.pone.0282090)
Supplement: S1 File — (DOCX) [file pone.0282090.s003.docx]

**S1 Text. The number of rooms/time points where sampling was not performed.** A bold font indicates missing data at all time points before or after the intervention, so this room could not be included in the analysis.

Sampling not performed:

^3^In 1 room in 1 out of 3 time points before the intervention, for both the inner and outer sink environment.

^4^1) in 2 rooms in 1 out of 3 time points before the intervention, for both the inner and outer sink environment, 2) in 2 rooms in 1 out of 3 time points after the intervention, for both the inner and outer sink environment, and 3) in 1 room in 1 out of 3 time points after the intervention, for only the inner sink environment.

^5^In 1 room in 1 out of 3 time points after the intervention, for both the inner and outer sink environment.

^6^1) in 3 rooms 1 out of 3 time points before the intervention, for only the outer sink environment, 2) in 1 room in 1 out of 3 time points before the intervention, for both the inner and outer sink environment, 3) in 1 room in 1 out of 3 time points after the intervention, for both the inner and outer sink environment.

^7^1) in 1 room in 1 out of 3 time points after the intervention, for both the inner and outer sink environment, 2) in 1 room in 1 out of 3 time points before the intervention, for only the outer sink environment.

^8^In 1 room in 1 out of 3 time points before the intervention, for only the outer sink environment.

^9^1) in 1 room in 1 out of 3 time points after the intervention, for only the inner sink environment, 2) in 4 rooms in 1 out of 3 time points before the intervention, for both the inner and outer sink environment, 3) in 2 rooms in 1 out of 3 time points before the intervention, for only the outer sink environment, 4) in 2 rooms 2 time points before the intervention, for both the inner and outer sink environment, 5) in 1 room 2 time points before, and 2 time points after the intervention, for both the inner and outer sink environment, 6) in **1 room all 3 time points before**, and 1 time point after the intervention, for both the inner and outer sink environment, 7) in **1 room all 3 time points before**, for both the inner and outer sink environment.

^10^1) in 1 room 1 time point after the intervention, for both the inner and outer sink environment, 2) in 1 room 1 time point before the intervention, for only the outer sink environment, 3) **in 3 rooms** 2 time points before the intervention, for both the inner and outer sink environment, and **all 3 time points after the intervention**, for both the inner and outer sink environment, 4) in **1 room** **all 3 time points before the intervention**, for both the inner and outer sink environment, and 1 time point after the intervention, for both the inner and outer sink environment.

^11^In 1 room 2 time points after the intervention, for both the inner and outer sink environment.

**S3 Figure.** **Time series figure of VIM-PA-positive samples found at every sampling moment before and after the intervention per sink surface.**

Abbreviations: W, weeks; M, months; VIM-PA, Verona Integron-encoded Metallo-beta-lactamase-positive *Pseudomonas aeruginosa*.

Y-axis, percentage of VIM-PA positivity; x-axis, time points before and after the intervention.

**S4 Table.** **Percentages of VIM-PA-positive samples in dirty utility rooms before and after the intervention.**

| **Sink environment** | **Sink surface** | **VIM-PA positive samples (%)** | |
| --- | --- | --- | --- |
|  |  | **Before (n=36)** | **After (n=35^1^)** |
| Outer | Countertop | 0 (0) | 0 (0) |
| Outer | Wash basin | 0 (0)^1^ | 3 (8.6) |
| Outer | Faucet aerator | 0 (0)^2^ | 0 (0)^4^ |
| Inner | Siphon water | 4 (12.9)^3^ | na |
| Inner | Drain | 15 (41.7) | 9 (25.7) |

Abbreviations: VIM-PA, Verona Integron-encoded Metallo-beta-lactamase-positive *Pseudomonas aeruginosa*; na, not applicable.

^1^One missing sample. ^2^16 missing samples. ^3^Five missing samples. ^4^Two missing samples.

**S5 Table.** **Time point analysis represented as a heat map.** Sinks in three rooms each at wards ICU-1 and Gastroenterology and Hepatology (six rooms total) were screened for *bla*_VIM_ for five consecutive days at 9:00, 12:30, and 16:00. Shown in the table are the number of days per room per sink surface with a VIM-PA-positive culture.

| **Sink environment** | **Sink surfaces** | **Room** | **Time** | | |
| --- | --- | --- | --- | --- | --- |
|  |  |  | **9:00** | **12:30** | **16:00** |
| Outer | Countertop | ICU-1, single-occupancy patient room 1 | 0 | 0 | 0 |
|  |  | ICU-1, single-occupancy patient room 2 | 0 | 0 | 0 |
|  |  | ICU-1; laboratory | 0 | 0 | 0 |
|  |  | G&H; medication room | 0 | 1 | 0 |
|  |  | G&H; 4-bed patient room | 0 | 0 | 0 |
|  |  | G&H; 2-bed patient room | 0 | 0 | 0 |
| Outer | Wash basin | ICU-1, single-occupancy patient room 1 | 0 | 0 | 0 |
|  |  | ICU-1, single-occupancy patient room 2 | 0 | 2 | 1 |
|  |  | ICU-1; laboratory | 0 | 0 | 0 |
|  |  | G&H; medication room | 3 | 2 | 4 |
|  |  | G&H; 4-bed patient room | 0 | 0 | 0 |
|  |  | G&H; 2-bed patient room | 0 | 3 | 0 |
| Outer | Faucet aerator | ICU-1, single-occupancy patient room 1 | 0 | 0 | 0 |
|  |  | ICU-1, single-occupancy patient room 2 | 0 | 0 | 0 |
|  |  | ICU-1; laboratory | 0 | 0 | 0 |
|  |  | G&H; medication room | 0 | 1 | 1 |
|  |  | G&H; 4-bed patient room | 0 | 0 | 0 |
|  |  | G&H; 2-bed patient room | 0 | 0 | 0 |
| Inner | Siphon water | ICU-1, single-occupancy patient room 1 | 2 | 2 | 2 |
|  |  | ICU-1, single-occupancy patient room 2 | 5 | 5 | 5 |
|  |  | ICU-1; laboratory | 0 | 0 | 0 |
|  |  | G&H; medication room | 5 | 4 | 5 |
|  |  | G&H; 4-bed patient room | 0 | 0 | 0 |
|  |  | G&H; 2-bed patient room | 4 | 3 | 3 |
| Inner | Drain | ICU-1, single-occupancy patient room 1 | 2 | 1 | 3 |
|  |  | ICU-1, single-occupancy patient room 2 | 4 | 5 | 5 |
|  |  | ICU-1; laboratory | 0 | 0 | 0 |
|  |  | G&H; medication room | 5 | 5 | 5 |
|  |  | G&H; 4-bed patient room | 0 | 0 | 0 |
|  |  | G&H; 2-bed patient room | 5 | 5 | 5 |

Abbreviations: G&H, Gastroenterology and Hepatology; ICU, intensive care unit.
